# Long-Term Sleep Deprivation-Induced Myocardial Remodeling and Mitochondrial Dysfunction in Mice Were Attenuated by Lipoic Acid and *N*-Acetylcysteine

**DOI:** 10.3390/ph16010051

**Published:** 2022-12-29

**Authors:** Fei Song, Jiale Lin, Houjian Zhang, Yuli Guo, Yijie Mao, Zuguo Liu, Gang Li, Yan Wang

**Affiliations:** 1 Xiamen Cardiovascular Hospital of Xiamen University, School of Medicine, Xiamen University, Xiamen 361000, China; 2 Eye Institute of Xiamen University, Fujian Provincial Key Laboratory of Ophthalmology and Visual Science, Fujian Engineering and Research Center of Eye Regenerative Medicine, Xiamen 361102, China; 3 Department of Ophthalmology, Xiang’an Hospital and Xiamen Eye Center Affiliated to Xiamen University, Eye Institute of Xiamen University, Fujian Provincial Key Laboratory of Ophthalmology and Visual Science, Fujian Engineering and Research Center of Eye Regenerative Medicine, Xiamen 361102, China

**Keywords:** sleep deprivation, mitochondria, myocardial remodeling, lipoic acid, *N*-acetylcysteine, reactive oxygen species

## Abstract

The impact of long-term sleep deprivation on the heart and its underlying mechanisms are poorly understood. The present study aimed to investigate the impact of chronic sleep deprivation (CSD) on the heart and mitochondrial function and explore an effective drug for treating CSD-induced heart dysfunction. We used a modified method to induce CSD in mice; lipoic acid (LA) and *N*-acetylcysteine (NAC) were used to treat CSD mice. Echocardiography, hematoxylin-eosin (H&E) staining, Sirius red staining, and immunohistochemistry were used to determine heart function and cardiac fibrosis. The serum levels of brain natriuretic peptide (BNP), superoxide Dismutase (SOD), micro malondialdehyde (MDA), and glutathione (GSH) were measured to determine cardiovascular and oxidative stress-related damage. Transmission electron microscopy was used to investigate mitochondrial damage. RNA-seq and Western blotting were used to explore related pathways. We found that the left ventricular ejection fraction (LVEF) and fraction shortening (LVFS) values were significantly decreased and myocardial hypertrophy was induced, accompanied by damaged mitochondria, elevated reactive oxygen species (ROS), and reduced SOD levels. RNA-sequence analysis of the heart tissue showed that various differentially expressed genes in the metabolic pathway were enriched. Sirtuin 1 (Sirt1) and Glutathione S-transferase A3 (Gsta3) may be responsible for CSD-induced heart and mitochondrial dysfunction. Pharmacological inhibition of ROS by treating CSD mice with LA and NAC effectively reduced heart damage and mitochondrial dysfunction by regulating Sirt1 and Gsta3 expression. Our data contribute to understanding the pathways of CSD-induced heart dysfunction, and pharmacological targeting to ROS may represent a strategy to prevent CSD-induced heart damage.

## 1. Introduction

Sleep is essential to human health and well-being and plays a vital role in an individual’s mental, emotional, and physiological health [1]. An increasing number of people experience insufficient sleep and chronic sleep deprivation. Sleep disruption is associated with increased activity of the sympathetic nervous system and hypothalamic–pituitary–adrenal axis, metabolic effects, changes in circadian rhythms, and pro-inflammatory responses [2]. Sleep disorders can also lead to adverse health outcomes. Decreased quantity and quality of sleep, whether due to sleep disorders or lack of proper sleep patterns, have been linked to cardiovascular disease (CVD) risk factors [3], such as hypertension, obesity, diabetes, and dyslipidemia [4,5,6]. Although the impact of sleep deprivation has been extensively studied in various literature, its mechanism is still not well understood, especially in investigating long-term chronic sleep deprivation (CSD).

Accumulating evidence has shown that sleep disorders induce multi-organ injury, including the liver, lung, kidney, and spleen, with oxidative stress and inflammation [7] and metabolic consequence [5,6,7]. In a proteomic analysis of rat serum of CSD (6 weeks) on metabolism, the authors identified four proteins (pyruvate kinase M1, clusterin, kininogen1, and profilin-1), as potential biomarkers for CSD in the myocardium and brain of a rat model [7]. Most of the previous basic studies focused on the outcomes of short-term sleep deprivation [3,8,9,10,11,12]. However, the influence of long-term CSD on heart function, oxidative stress, and the molecular pathways involved in CSD remain unclear.

In our present study, we employed a modified “stick over water” method [13] to establish the CSD mouse model for four months. Compared to other methods, such as the rotating drum model and gentle stimulation model, our present CSD method is simple and easy to operate, no complex equipment is needed, and the process can be observed for a long time, which is more conducive to long-term modeling. We applied the modified CSD model to explore the impact of long-term sleep deprivation on heart function, myocardial fibrosis, myocardial hypertrophy, myocardial mitochondrial function, oxidative stress, and related molecular pathways. We then applied two antioxidants, α-lipoic acid (LA) and *N*-acetylcysteine (NAC), to explore their potential treatment effects on CSD.

## 2. Results

### 2.1. Chronic Sleep Deprivation Decreased Cardiac Function, Induced Hypertrophic Cardiomyopathy, and Myocardial Fibroblast

**Figure 1:**
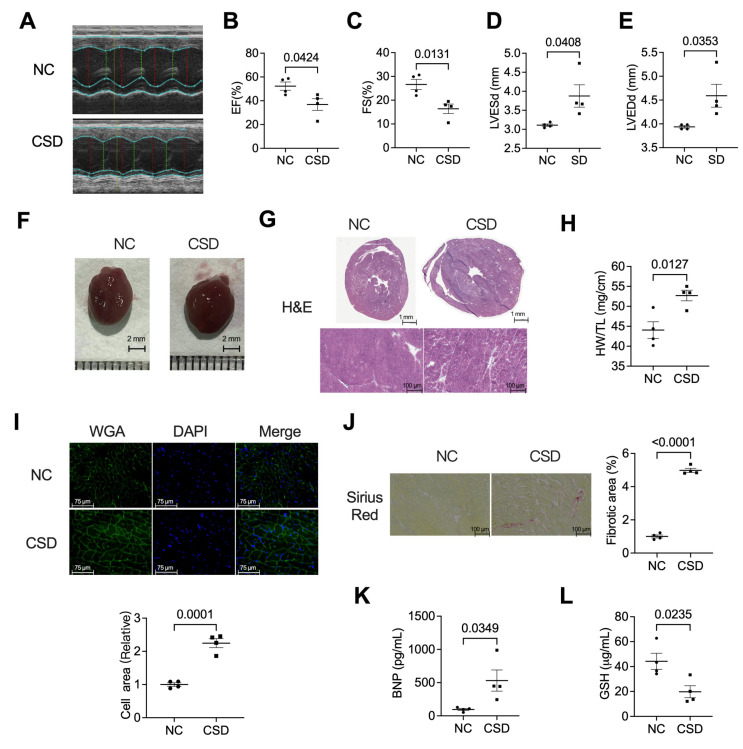
CSD led to myocardial hypertrophy and heart failure in adult mice. (**A**) Representative echocardiogram and analysis of (**B**) left ventricular (LV) Eject Fraction (EF), (**C**) Fraction Shortening (FS), (**D**) left ventricle end-systolic diameter (LVESd), and (**E**) left ventricle end-diastolic diameter (LVEDd). (**F**) Representative anatomic images of control (NC) and chronic sleep deprivation (CSD) mice (scale bar = 2 mm). (**G**) Hematoxylin & eosin (H&E) images of crosscut section heart of control and CSD mice (scale bar = 1 mm or 100 μm). (**H**) The heart weight (HW) ratio to tibia length (TL) with or without four months of CSD. (**I**) Representative image and analyzed data of immunostaining of WGA indicating the cardiomyocyte size of NC and CSD. (**J**) Representative images and analyzed data of Sirius red-stained hearts showed increased collagen deposition in CSD hearts. The fibrotic regions (Red) in six different fields per heart (four hearts per group) were measured by ImageJ (scale bar = 100 μm). Serum levels of GSH (**K**) and BNP (**L**) in NC and CSD mice.

We generated a murine model of chronic sleep deprivation to investigate the impact of long-term sleep deprivation on the heart. After four months of sleep deprivation, an echocardiographic evaluation was performed to determine cardiac function (Figure 1A–E). The results showed that the EF value decreased from more than 50% in the regular sleep group to less than 40% in the CSD group (Figure 1B). Similar to the EF value, the FS value (Figure 1C) also decreased in CSD mice. However, the LVESd (Figure 1D) and LVEDd (Figure 1E) increased. We then investigated the hypertrophic phenotype in CSD mice hearts, as shown in Figure 1F through Figure 1I. The heart size (Figure 1F,G), heart weight/tibia length ratios (Figure 1H), and cross-sectional area of cardiomyocytes (Figure 1I) of CSD mice were substantially higher than those of control mice. In addition, CSD induced cardiac fibrosis, as shown by picrosirius red staining of heart sections (Figure 1J). Serum levels of BNP (Figure 1K) and GSH (Figure 1L), which are related to cardiac damage, were also upregulated by CSD induction. Long-term chronic sleep deprivation can result in significant cardiac dysfunction and hypertrophy.

### 2.2. Chronic Sleep Deprivation Accumulated ROS and Induced Mitochondrial Dysfunction in the Heart

**Figure 2:**
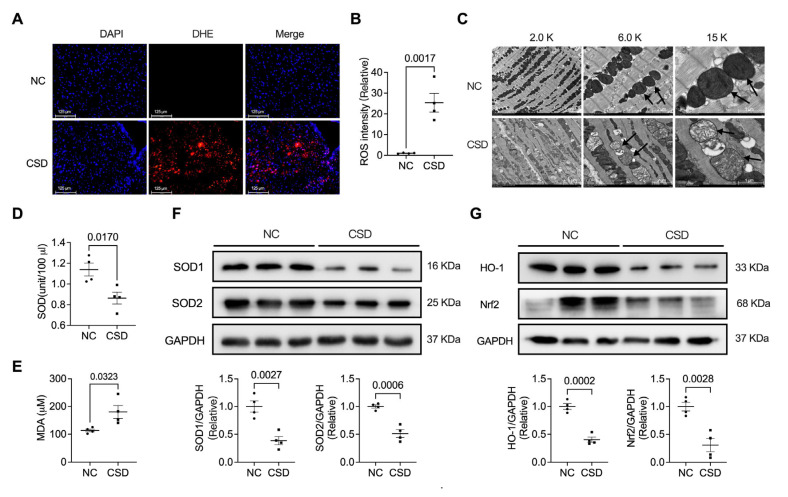
CSD induced mitochondrial impairment and elevated oxidative stress in the heart. (**A**) DHE staining and analysis (**B**) unravels increased lipid peroxidation in the CSD heart, indicating elevated oxidative stress. DHE fluorescent intensity was measured by ImageJ (scale bar = 125 μm). (**C**) Electron micrographs showing mitochondria in heart tissue obtained from control (NC) and chronic sleep deprivation (CSD) mice (scale bar = 5 μm, 2 μm, 1 μm). Serum levels of SOD (**D**) and MDA (**E**) in NC and CSD mice. (**F**) Representative Western blot image and quantification of SOD1 and SOD2 in NC and CSD hearts. (**G**) Representative Western blot image and quantification of HO-1 and Nrf2 in NC and CSD hearts.

To further explore the influence of CSD on cardiac dysfunction, we examined ROS levels and mitochondrial-related phenotypes. CSD significantly promoted cardiac ROS production (Figure 2A,B). ROS overload can induce mitochondrial dysfunction. We then observed mitochondrial structure using TEM. We found that the structure of mitochondria was destroyed by long-term sleep deprivation (Figure 2C). Moreover, SOD (Figure 2D) in the blood and the expression of SOD1 and SOD2 (Figure 2F) were significantly decreased, and the serum level of MDA (Figure 2E) was increased in CSD-exposed mice. The levels of mitochondria-related proteins HO-1 and Nrf2 were also reduced in the heart tissues of CSD mice (Figure 2G).

### 2.3. Sirt1 and Gsta3 Were Involved in CSD-Induced Cardiac Dysfunction

**Figure 3:**
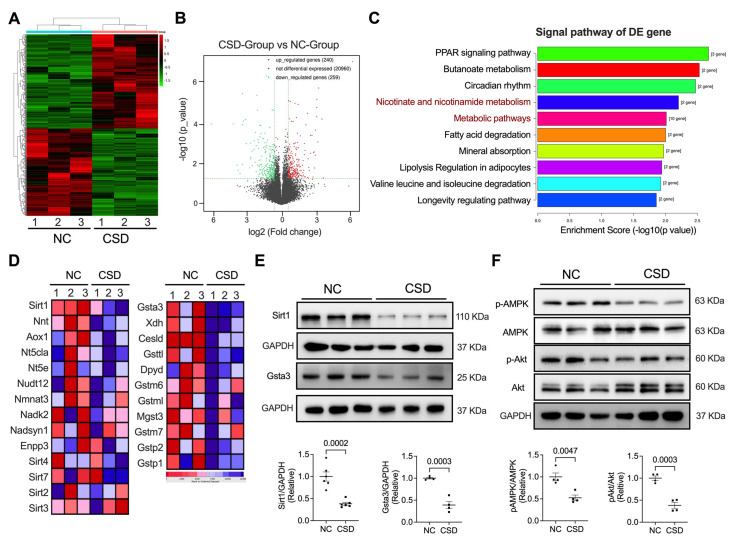
RNA sequencing revealed the Sirt1−Gsta3 axis is involved in CSD−regulated heart dysfunction. (**A**) RNA sequencing heat map clustered in hierarchy volcano map (**B**) shows up− or down−regulated genes in NC and CSD hearts. (**C**) Signal pathway analysis of the differential expression (DE) gene of RNA−seq shows the top 10 pathways related to CSD treatment. (**D**) Heat map clustered in hierarchy shows that Sirt1 of the Nicotinate and nicotinamide metabolism pathway (Left panel) and Gsta3 of the metabolic pathway were downregulated in the CSD heart. (**E**) Representative Western blot images and quantification of Sirt1 and Gsta3 in NC and CSD hearts. (**F**) Representative Western blot images and quantification of phosphorylation or total of AMPK and Akt in NC and CSD hearts.

To identify the molecular mechanisms underlying the development of heart failure in CSD mice, we performed RNA-seq analyses in both control (NC) and CSD mice. CSD promoted 240 upregulated and 259 downregulated genes (Figure 3A,B). Several molecular players involved in store-operated and receptor-operated calcium entry and the hypertrophy reaction are shown in Figure S1. Interestingly, analysis of differentially expressed genes suggested that both nicotinate and nicotinamide metabolism and metabolic pathways are critical pathways that participate in CSD-induced heart dysfunction. Among the two pathways, Sirt1 and Gsta3 were the most downregulated genes (Figure 3C,D). We then verified the expression of Sirt1 and Gsta3 in the heart at the protein level and found that they were also reduced after CSD induction (Figure 3E). AMPK and AKT are essential pathways for regulating cell metabolism and fate. Interestingly, we found that the phosphorylation of AMPK and Akt was downregulated in CSD hearts compared to that in control hearts (Figure 3F).

### 2.4. Administration of Antioxidants Ameliorated CSD-Induced Dysfunction of and Hypertrophic Cardiomyopathy

**Figure 4:**
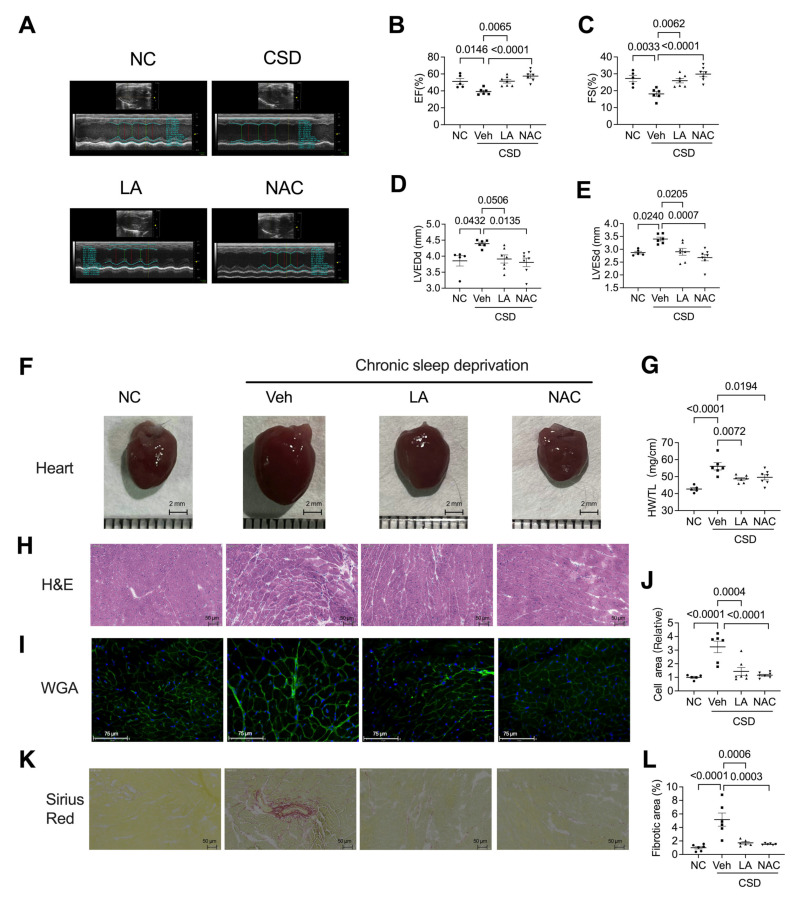
Antioxidants significantly rescue CSD-induced heart failure and myocardial hypertrophy. Representative echocardiography (**A**) and quantification of EF (**B**), FS (**C**), LVEDd (**D**), and LVESd (**E**) of the four indicating groups. (**F**) Representative anatomic images of four indicating groups (scale bar = 1 mm). (**G**) The ratio of heart weight (HW) to tibia length (heart/tibia) of four indicated groups. (**H**) Hematoxylin & eosin (HE) images of crosscut section heart of four displaying groups. Representative image (**I**) and quantification (**J**) of immunostaining of WGA indicating the cardiomyocyte size of four indicating groups (scale bar = 75 μm). Representative images (**K**) and analyzed data (**L**) of Sirius red-stained hearts of four indicating groups (scale bar = 50 μm).

To explore the role of antioxidants in CSD-induced heart damage, we employed two antioxidants, LA and NAC, and administered them with CSD induction. As shown in Figure 4, LA and NAC significantly rescued heart functions, such as EF and FS values (Figure 4A–C). LA and NAC also decreased hypertrophic cardiomyopathy, both in the echocardiography in LVEDd and LVESd (Figure 4D,E) and morphological observation (Figure 4F,G), heart weight/tibia length ratios (Figure 4H), and WGA staining (Figure 4I,J). In addition, CSD-induced myocardial remodeling in the perivascular region was reversed by antioxidants (Figure 4K,L).

### 2.5. Antioxidants Protected against Mitochondria Dysfunction Induced by CSD

**Figure 5:**
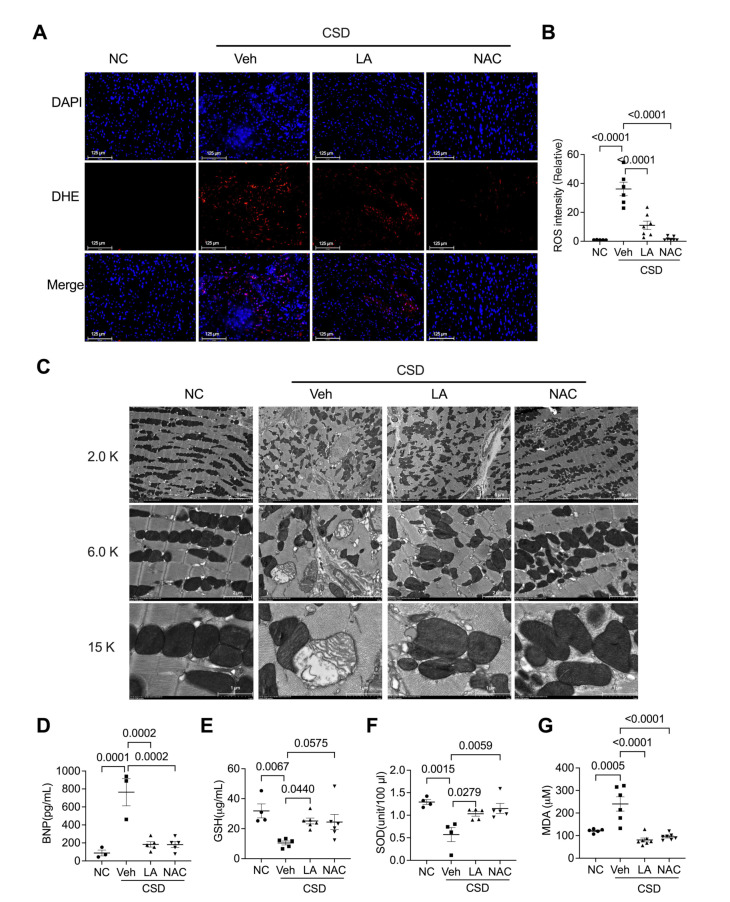
Antioxidants alleviated CSD-induced impaired mitochondria. (**A**) DHE staining images and analyzed data (**B**) unravel increased lipid peroxidation heart of four indicating groups (scale bar = 125 μm). (**C**) Representative transmission electron microscopy (TEM) images of the heart of four indicated groups (scale bar = 5 μm, 2 μm, 1 μm). Serum levels of BNP (**D**), GSH (**E**), SOD (**F**), and MDA (**G**) in four indicating groups.

We then investigated ROS- and mitochondria-related changes in antioxidant-treated hearts. The results showed that both LA and NAC significantly reversed CSD-induced increases in ROS levels (Figure 5A,B). The damaged mitochondrial structure was also rescued by oral administration of LA or NAC (Figure 5C). Moreover, LA and NAC significantly decreased the CSD-induced increases in BNP and MDA levels (Figure 5D,E). The blood levels of GSH and SOD were also reversed by LA and NAC (Figure 5F,G).

### 2.6. Antioxidants Reversed CSD-Induced Cardiac Dysfunction by Regulating the Nrf2/Sir1/Gsta3 Axis

**Figure 6:**
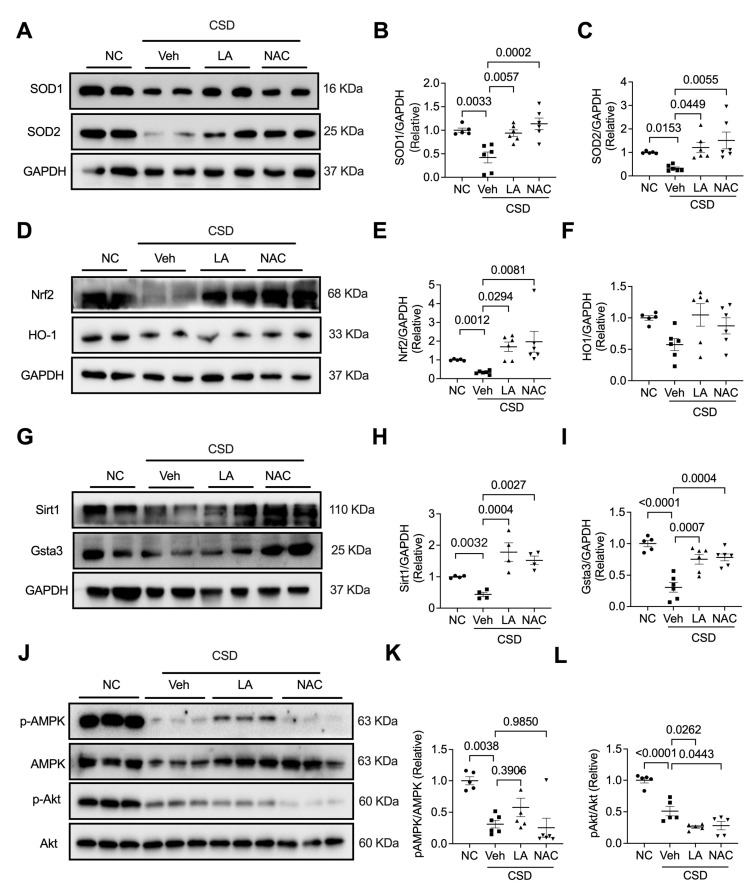
Antioxidants regulated Sirt1/Gsta3/SODs signaling pathway in CSD-induced heart failure. Representative western blot images and quantification of SOD1 (**A**,**B**), SOD2 (**A**,**C**), Nrf2 (**D**,**E**), HO1 (**D**,**F**), Sirt1 (**G**,**H**), Gsta3 (**G**,**I**), and the phosphorylation of AMPK (**J**,**K**) and Akt (**J**,**L**) in four indicating hearts.

To further explore the molecular pathways involved in the two antioxidants, LA and NAC, in CSD-induced cardiac dysfunction, we examined the expression of SOD1, SOD2, Nrf2, HO-1, Sirt1, and Gsta3 and the phosphorylation of AMPK and Akt. As shown in Figure 6, CSD-decreased SOD1 and SOD2 levels were effectively promoted by treatment with LA and NAC. Interestingly, CSD-restrained expression of Nrf2, Sirt1, and Gsta3 was neutralized by LA and NAC treatment. However, LA and NAC had no significant effects on the CSD-induced decrease in HO-1 expression (Figure 6D,F) or phosphorylation of AMPK and Akt (Figure 6G–I). These results highlight a clear pathway through which LA and NAC regulate CSD-induced heart dysfunction via the Nrf2/Sir1/Gsta3 pathway.

## 3. Discussion

There were a series of significant findings in this study. First, long-term chronic sleep deprivation (CSD) induces cardiac dysfunction, myocardial hypertrophy, and myocardial fibrosis, accompanied by decreased serum GSH levels and increased BNP levels. Second, CSD-exposed mice showed mitochondrial dysfunction by elevated ROS production, reduced serum SOD and myocardial protein levels of SOD1 and SOD1, and restrained HO-1 and Nrf2. Third, the RNA sequence of heart tissues revealed that Sirt1 and Gsta3 are the critical pathways that participate in long-term chronic sleep deprivation-induced cardiac dysfunction. Fourth, the administration of antioxidants LA and NAC can effectively rescue CSD-induced cardiac dysfunction, myocardial hypertrophy, mitochondrial dysfunction, and myocardial fibrosis accompanied by increased serum levels of GSH and SOD and decreased ROS production in the heart and serum levels of BNP and MDA. Finally, Sirt1 and Gsta3 may be the main pathways involved in the antioxidant effect on CSD-induced myocardial dysfunction.

Sleep is an essential and fundamental physiological process that plays a crucial role in balancing the psychological and physical health of almost all animals [12]. Previous studies have shown that reduced total sleep time is related to increased cardiovascular risk [3,7,9,11,14,15,16]. The recent guidelines for cardiovascular prevention released by the American College of Cardiology/American Heart Association (2019) state that counseling on sleep and sleep hygiene (along with advice on physical activity) should be provided to prevent cardiovascular diseases (CVD) [17]. Most previous animal and human studies on the impact of sleep deprivation on the heart have focused on short-term influences [3,8,9,10,18,19,20,21]. Few studies have demonstrated the impact of long-term sleep deprivation on heart function and mitochondria in experimental animals. A previous study investigated the serum proteomic analysis in 6-week chronic sleep deprivation and found that four proteins, including pyruvate kinase M1, clusterin, kininogen1, and profilin-1, which are related to energy metabolism, cardiovascular function, and nerve function, were identified as potential biomarkers for chronic sleep deprivation [7]. In the present study, long-term chronic sleep deprivation induced significant structural and functional echocardiographic abnormalities. Long-term chronic sleep deprivation decreases heart function by decreasing left ventricular EF, FS, and SOD, promoting BNP and GSH, and ROS production, and damaging the mitochondrial structure. SOD is a critical antioxidant enzyme that catalyzes the conversion of superoxide to hydrogen peroxide and molecular oxygen [22,23]. Defects in SOD levels may result in severe heart dysfunction. In a previous in vivo study on chronic sleep restriction, the authors found frontal cortical mitochondrial dysfunction and mitochondria-related β-amyloid accumulation [24], which coincided with our results on mitochondria in CSD-exposed hearts. ROS plays a crucial role in the development of cardiovascular complications. An imbalance between ROS generation and antioxidant capacity, favoring the former, leads to oxidative stress and damage [25]. Our results showed that CSD in mice induced severe elevation of ROS production in mouse hearts, which may be one of the crucial factors for cardiac and mitochondrial dysfunction. After a long time of sleep deprivation induction, cardiac fibrosis was induced. It is reported that the accumulation of ROS triggers a wide range of pro-fibrogenic signals, including cellular inflammatory cytokines, transcription factors, stress reactive protein kinase, and so on, which further promotes the activation of fibroblasts to promote cardiac fibrosis [26,27,28]. Moreover, mitochondrial dysfunction would accelerate the production of ROS and the cytoplasmic release of cytochrome, which enables cardiomyocyte injury, and programmed cell death and finally results in heart failure and cardiac fibrosis [29,30,31]. CSD-induced ROS accumulation and mitochondrial dysfunction may be the potential reason for cardiac fibrosis.

*N*-acetylcysteine (NAC) is a sulfur-containing amino acid found in onions, garlic, and protein-rich food [32]. It prevents DNA damage, inhibits oxidative stress, and increases intracellular levels of glutathione. NAC is mainly used in the clinic as a mucolytic agent in the treatment of pulmonary diseases and other indications, including acetaminophen overdose [33], and contrast-induced nephropathy [34]. NAC is a favorable source of L-cysteine, which has been suggested to regulate diverse pathways, such as oxidative stress via the production of GSH and taurine [35]. Compared to cysteine, NAC has advantages such as tolerability, water solubility, and less susceptibility to oxidation [36]. Our results showed that pharmacological use of NAC in CSD-exposed mice was able to revert ROS production, GSH, and MDA, and rescue changed the mitochondrial structure and heart function. These data are in line with previous studies reporting the beneficial effects of NAC against oxidative damage in cardiac tissue induced by cisplatin [37] or isoprenaline [32].

Like NAC, α-Lipoic acid (LA) is another potent antioxidant used worldwide, mainly due to the disulfide group of its molecular structure, which has the ability to react with ROS [38]. Another antioxidant feature of LA is its ability to chelate transition metals and avoid ROS formation [39]. LA is an endogenous fatty acid and a regulator of energy metabolism in the mitochondria [40]. Owing to its antioxidant properties, LA is protective in various pathologic cardiovascular conditions, such as atherosclerosis [41,42,43], hypertension [44,45,46], heart failure [47,48], and diabetes-related cardiovascular disorders [39,49,50]. In this context, we demonstrated the novel ability of LA and NAC to treat sleep deprivation-induced cardiac dysfunction and mitochondrial damage. Interestingly, CSD-induced mitochondrial damage was also restored by the antioxidants NAC and LA, suggesting that CSD-induced injury in heart tissue may be partially protected by increasing these antioxidants via the regulation of mitochondrial damage. The main molecular pathways of the two antioxidants promoted the expression of SOD1, SOD2, and Nrf2 and increased the Sirt1 and Gsta3 pathways.

In summary, the present data highlight long-term sleep deprivation-induced cardiac dysfunction, myocardial hypertrophy, and cardiovascular cardiomyopathy complications. Another highlight of the present study is the importance of antioxidant compounds as protective agents against chronic sleep disorder-related cardiac dysfunction. The findings herein also show that ROS and MDA consumption increased after four months of sleep deprivation, and supplementation with NAC and LA restored the increased levels of these antioxidants. Concomitantly, serum SOD and expression of SOD1 and SOD2 in the heart decreased after sleep deprivation, and NAC and LA reversed these effects. The main pathway regulated by CSD-induced mitochondrial dysfunction and oxidative stress in the heart may be mediated by Sirt1 and Gsta3. NAC and LA presented the most evident antioxidant effects, decreasing protein levels and mitochondrial damage. Considering the necessary precautions, it is possible to suggest that NAC and LA supplementation showed a beneficial antioxidant effect and may contribute to preventing cardiovascular diseases induced by sleep disorders. Additionally, reducing CSD may be the best advice for patients in this setting. The main limitation of our study could be the lack of data regarding in vitro studies since there is no effective and valuable model for in vitro research on sleep deprivation. The model requires further functional exploration in terms of neurological changes, such as in different brain monoaminergic pathways, endocrine changes (stress hormones like catecholamines and cortisol, vasopressin, renin-angiotensin-aldosterone, insulin/glucagon, etc.), and peripheral effects on different target organs.

## 4. Materials and Methods

### 4.1. Induction of Chronic Sleep Deprivation

All animal experiments were approved by the Committee of Xiamen University and were carried out following the guidelines for the protection and use of laboratory animals of the National Institute of Health (NIH). Adult male C57BL/6 mice (6–8 weeks old, weight: 20–25 g) were purchased from the Shanghai SLAC Laboratory Animal Center (Shanghai, China) and raised in the Specific pathogen-free (SPF) Experimental Animal Center of Xiamen University. Eight mice were used to test the effect of CSD on the heart. We applied a modified “stick over water” method [13] to establish a CSD mouse model for four months. Briefly, two circular wooden sticks (6 mm diameter) were placed across the sidewalls of the cage at a height of 4.0 cm from the bottom. The cages were filled with water to a level of 1.0 cm beneath the sticks. The horizontal distance between the two sticks was 6.0 cm, which allowed the mice to move between them. The mice had unrestricted access to food and water while standing on the front stick. When the mice fell asleep while standing on the stick, muscle atony caused the animal to lose balance and slip down to the water’s surface, which awakened the animal. Mice in the CSD group (N = 4) were placed on this stick configuration for 20 h (19:00–15:00) per day and transferred to their home cages during the resting phase (15:00–19:00). Before the experiment, each mouse was adapted to the CSD procedure for one h on three consecutive days. Two mice were housed in each cage. Control mice (N = 4) were housed in standard cages. CSD and control mice were maintained for four months.

### 4.2. Antioxidant Feeding

To investigate whether antioxidants can rescue CSD-induced myocardial dysfunction and oxidative stress, we used α-lipoic acid (LA, Catalog# HY-18733, MCE, South Brunswick, NJ, USA) and *N*-acetylcysteine (NAC, Catalog# HY-B0215, MCE, South Brunswick, NJ, USA) in the following study. A total of 25 C57BL/6 mice were randomly divided into four groups: control group (NC, N = 5), CSD group (CSD, N = 6), CSD with α-Lipoic acid treatment group (CSD + LA, N = 7), and CSD with *N*-acetylcysteine (NAC) treatment group (CSD + NAC, N = 7). For the LA and NAC treatments, the two antioxidant compounds were diluted in the appropriate solvent, with Dimethyl sulfoxide (DMSO) or H_2_O, and added to cornmeal-agar food at the following final concentrations: LA, 100 mg (Dissolved in 2 mL DMSO)/kg (cornmeal-agar food); NAC, 500 mg (Dissolved in H_2_O)/kg (cornmeal-agar food). In the vehicle group (Veh), cornmeal-agar food was crushed and added with 2 mL/kg DMSO and H_2_O. The mice in the NC groups were fed cornmeal agar. Animals were administered LA or NAC at the start of the CSD model for four months.

### 4.3. Echocardiography Evaluation

Echocardiography was performed to detect the heart function of the mice using a Visual Sonics Vevo2100 (Visual Sonics, Toronto, ON, Canada) imaging system at the end of the experiment. Briefly, mice were anesthetized with 3% isoflurane and maintained with 2% isoflurane with a nosecone on a heated platform. Heart rate and left ventricular dimensions, including diastolic and systolic wall thickness, left ventricle end-diastolic diameter (LVEDd), and left ventricle end-systolic diameter (LVESd), were recorded when the heart rate was maintained at 330–380 bpm from the 2D short-axis under M-mode tracings at the level of the papillary muscle. Left ventricular mass and functional parameters, including fractional shortening (FS), left ventricular ejection fraction (EF), and LV volume, were calculated using the above-mentioned primary measurements and accompanying software.

### 4.4. Hematoxylin-Eosin (H&E) Staining

After four months of CSD induction and antioxidant treatment, the mice were euthanized under deep anesthesia with isoflurane, and blood and heart were collected. Part of the heart was fixed in a tissue-freezing medium with optimal cutting temperature (OCT) compound, frozen in liquid nitrogen for a few seconds, stored at −80 °C, and sectioned 6 μm thick for the experiment. An H&E staining kit (Catalog #G1120, Solarbio, Beijing, China) was used to assess myocardial hypertrophy. The sections were fixed in 4% paraformaldehyde, washed with ddH_2_O, stained with hematoxylin, differentiated in 1% HCl-ethanol, soaked in ammonia water until the nucleus color became blue, and stained with eosin. Finally, tissues were dried for 48–72 h and photographed under a light microscope.

### 4.5. Picrosirius Red Staining

To measure myocardial collagen deposits, heart sections were stained with Sirius red using a Picrosirius Red Stain Kit (Catalog#Ab150681, Abcam, Cambridge, Cambs, UK) following the manufacturer’s instructions. Briefly, heart sections were soaked in 0.2% phosphomolybdic acid for 3 min, dyed on the 0.1% picrosirius red staining for 90 min, and photographed under a light microscope. The fibrosis area was calculated using the ImageJ software (NIH, Bethesda, MD, USA).

### 4.6. ROS Determination

Dihydroethidium (DHE, Catalog#S0063, Beyotime Biotech, Beijing, China) was used to stain heart sections to determine ROS levels. The mouse hearts were collected and fixed in OCT, immediately frozen in liquid nitrogen for a few seconds, stored in a −80 °C refrigerator, and sectioned to the 6 μm thickness of the experiment. Heart sections were incubated with 10 μm DHE at 37 °C for 20 min in the dark. After washing with PBS, the sections were re-stained with 2-(4-Amidinophenyl)-6-indolecarbamidine dihydrochloride (DAPI) solution (Catalog# C0060, Solarbio, Beijing, China) for nuclear staining and captured using a confocal microscope (TCS SP5, Leica, Wetzlar, Germany) to obtain fluorescence images. The intensity of ROS fluorescence was quantified using the ImageJ software (NIH, Bethesda, MD, USA).

### 4.7. Immunofluorescence Staining

The cardiomyocyte cross-sectional area was determined by staining with FITC-conjugated wheat germ agglutinin (WGA, Catalog #L4895; Sigma-Aldrich, St. Louis, MO, USA). The heart sections were fixed with acetone for 15 min on ice, then blocked in normal goat serum for 60 min after permeabilization with 0.3% Triton X-100 in PBS for 10 min, and then sections were incubated with FITC-conjugated WGA for 1 h. Finally, the DAPI solution was used to stain the nucleus.

### 4.8. Measurement of Serum Levels of BNP, GSH, SOD, and MDA

Serum levels of brain natriuretic peptide (BNP), glutathione (GSH), superoxide dismutase (SOD), and lipid peroxidation malondialdehyde (MDA) were measured using a Mouse BNP ELISA Kit (Catalog#M0204c, Elabscience, Wuhan, China), a GSH ELISA Kit (Catalog#0026C, Elabscience, Wuhan, China), Cu/Zn-SOD and Mn-SOD Assay Kit with WST-8 (Catalog#S0103, Beyotime Biotech, Beijing, China), and a Peroxidation MDA Assay Kit (Catalog#S0131S, Beyotime Biotech, Beijing, China), respectively, according to the manufacturer’s instructions.

### 4.9. RNA-Sequencing

Half of the cardiac apex of the NC (N = 3) and CSD (N = 3) groups was extracted, and about 1–2 ug RNA from each sample was extracted. The samples of the NC and CSD groups are individual experiments. cDNA libraries were constructed using the KAPA Stranded RNA-Seq Library Prep Kit (Illumina, San Diego, CA, USA) according to the manufacturer’s instructions. Gene expression profiling was performed using an Illumina NovaSeq 6000 instrument (Illumina). Differentially expressed genes (DEGs) identified from pairwise comparisons must match the selection criteria: the relevant adjusted *p* < 0.05. The threshold was set as >1.5 times difference, *p* ≤ 0.05, and FPKM (Fragments Per Kilobase of gene/transcript model per Million mapped fragments) ≥0.5 was used to screen differentially expressed genes and transcripts.

### 4.10. Western Blot Analysis

The frozen heart tissues from different groups were lysed by RIPA with 1% protease and phosphatase inhibitors (Roche, Basel, Switzerland) for Western blot assay. The appropriate amounts of samples were loaded and separated by 10% or 12% SDS-PAGE and transformed onto the PVDF membrane. Protein expression was detected using primary antibodies followed by HRP-conjugated secondary antibodies. Signals were detected using an enhanced chemiluminescence kit (GE Healthcare, Chicago, IL, USA) and captured using a chemiluminescence detection system (Flour Chem E, ProteinSimple, San Jose, CA, USA). Band densitometry was performed using ImageJ software (NIH, USA). Some of the primary antibodies, such as Anti-SOD1 antibody (Catalog# ab51254, Abcam), anti-heme oxygenase 1 (HO-1) (Catalog# ab52947, Abcam), Anti-SIRT1 antibody (Catalog# ab110304, Abcam), Anti-Nrf2 antibody (Catalog#ab92946, Abcam), and Anti-Keap1 antibody (Catalog#ab227828, Abcam) were purchased from Abcam. Anti-SOD2 (Catalog#sc-137254; Santa Cruz Biotechnology, Dallas, TX, USA) was obtained from Santa Cruz Biotech. GSTA3 antibody (Catalog# DF12624, Affinity, Changzhou, China) was purchased from Affinity. Anti-GAPDH (D16H11) (Catalog#5174, CST, Danvers, MA, USA), anti-AMPKα (Catalog#2532, CST, USA), anti-phospho-Akt (Ser473) (Catalog#4060, CST, USA), anti-Akt (Catalog#9272, CST), and anti-phospho-AMPKα (Thr172) (Catalog#2535, CST, USA) antibodies were purchased from CST.

### 4.11. Transmission Electron Microscopy

For transmission electron microscopy assay of mitochondrial structure, three hearts from each group were perfused with 2% formaldehyde, 2.5% glutaraldehyde in 0.15 M phosphate buffer pH 7.4, followed by 1% OsO4, 1.5% potassium ferrocyanide, and stained with 1% uranyl acetate. Thin sections were stained with uranyl and lead, and micrographs were obtained using a Hitachi HT-7800 electron microscope (Hitachi, Tokyo, Japan).

### 4.12. Data Analysis

All data are expressed as mean ± SEM and were analyzed using GraphPad Prism v9.0 (GraphPad Software, San Diego, CA, USA). The experiments in this study, such as an echocardiogram, histology, ELISA, and Western blotting, were performed by a professional researcher blind to the group conditions. An observer blind quantified the studies to the experimental groups. The dots represent the number of independent experiments in different mice. The numbers represent biological replicates. At least three independent experiments with two to four samples/experiments/groups were performed for each assay. Normally distributed data were analyzed using the unpaired, 2-tailed Student *t*-test (two groups) or 1-way ANOVA followed by the Tukey multiple comparisons test. For data that did not follow a normal distribution, the unpaired 2-tailed Mann–Whitney U test (two groups) or the Kruskal–Wallis test (three or more groups), followed by the Dunn post hoc test, was used. Statistical significance was set at *p* < 0.05.

## Data Availability

The data supporting the findings of this study are available from the corresponding author upon reasonable request.

## Supplementary Materials

The following supporting information can be downloaded at: https://www.mdpi.com/article/10.3390/ph16010051/s1, Figure S1.
