# Supplementary figures and images for: Long-Term Sleep Deprivation-Induced Myocardial Remodeling and Mitochondrial Dysfunction in Mice Were Attenuated by Lipoic Acid and N-Acetylcysteine
Source: Pharmaceuticals (Basel). 2022 Dec 29;16(1):51. doi: 10.3390/ph16010051 (PMC9866495; doi:10.3390/ph16010051)

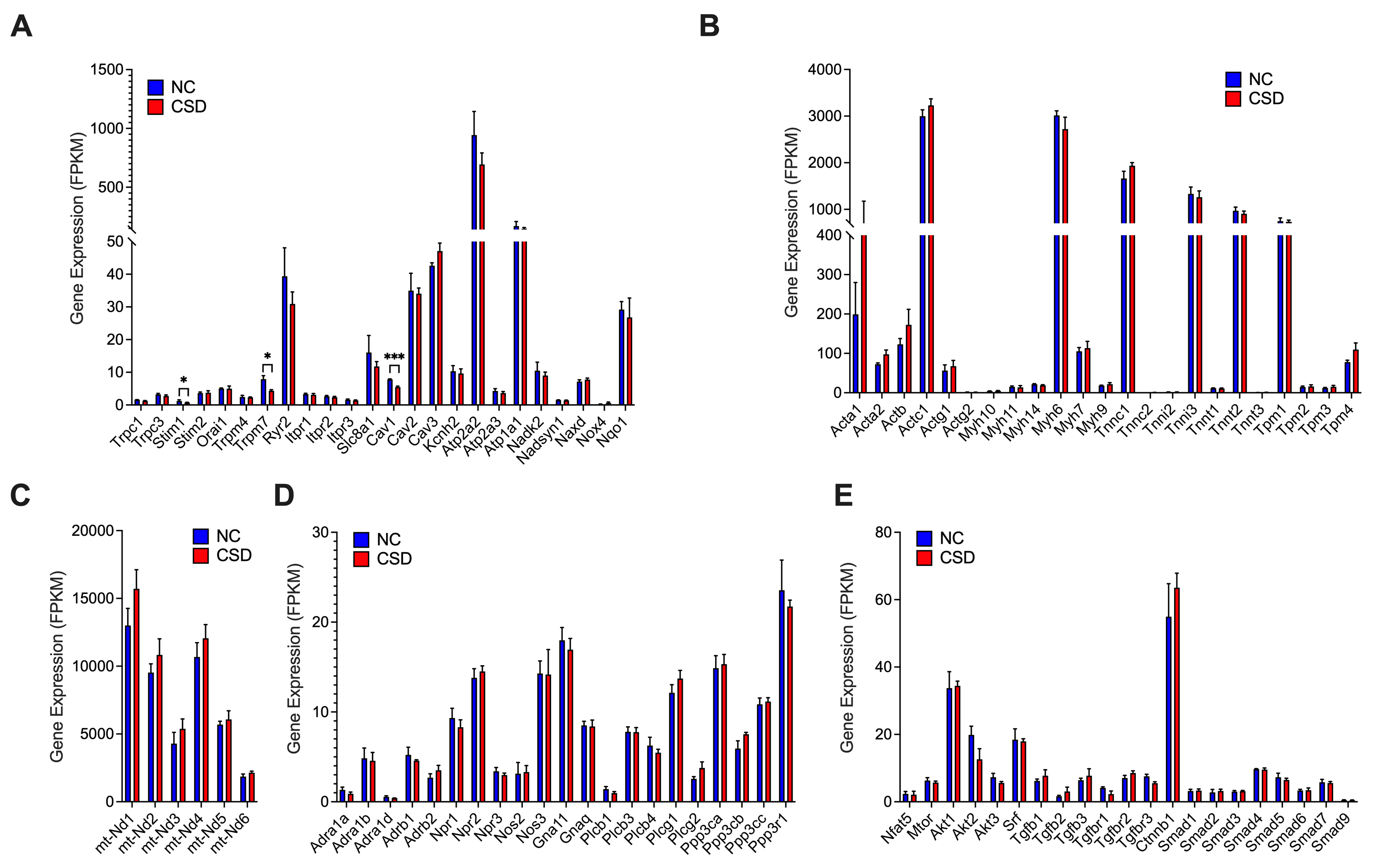

Supplement: Supplementary file 1 [file pharmaceuticals-16-00051-s001.zip › pharmaceuticals-2026737-supplementary.tiff]
